# Heterografted chrysanthemums enhance salt stress tolerance by integrating reactive oxygen species, soluble sugar, and proline

**DOI:** 10.1093/hr/uhac073

**Published:** 2022-03-23

**Authors:** Wenjie Li, Rui Meng, Ye Liu, Sumei Chen, Jiafu Jiang, Likai Wang, Shuang Zhao, Zhenxing Wang, Weimin Fang, Fadi Chen, Zhiyong Guan

## Abstract

Chrysanthemum, one of the most important commercial ornamental crops, is susceptible to salinity, which limits its cultivation and application in coastal and inland saline areas. Grafting is widely used to improve the salt tolerance of horticultural crops, but the mechanisms of grafted chrysanthemum responses to salt stress remain unclear. In this study, we showed that heterografted chrysanthemums with *Artemisia annua* as rootstock exhibited increased salt tolerance compared with self-grafted and self-rooted chrysanthemums. Under high salt stress, the roots of heterografted chrysanthemums enrich Na^+^, resulting in a reduction of Na^+^ toxicity in the scion, with only a small amount of Na^+^ being transported to the leaves. On the other hand, the roots of heterografted chrysanthemums alleviated high Na^+^ stress via enhanced catalase enzyme activity, downregulation of the expression of reactive oxygen species (ROS) accumulation-related genes, massive accumulation of soluble sugars and proline, and upregulation of the expression of heat shock protein-related genes to enhance salt tolerance. In addition, the leaves of heterografted chrysanthemums respond to low Na^+^ stress by increasing peroxidase enzyme activity and soluble sugar and proline contents, to maintain a healthy state. However, self-grafted and self-rooted plants could not integrate ROS, soluble sugars, and proline in response to salt stress, and thus exhibited a salt-sensitive phenotype. Our research reveals the mechanisms underlying the increased salt tolerance of heterografted chrysanthemums and makes it possible to have large-scale cultivation of chrysanthemums in saline areas.

## Introduction

Salinity affects >6% of the world’s total land area, ~800 million hectares of land [1]. Salt stress is a serious constraint on plant growth, development, and yield, and soil salinization is one of the major problems in modern agriculture [2]. High concentrations of Na^+^ and Cl^−^ in the soil create hyperosmotic conditions that can severely affect plant uptake of nutrients and water, increase toxic ion concentrations, and degrade soil structure [3, 4].

To resist salt stress and adapt to survival environment, plants produce a range of changes at the physiological, cellular, and molecular levels to regulate osmotic and ion homeostasis to maintain their development and normal growth [5], such as changes in Na^+^ efflux [6, 7], antioxidant enzymes [8], osmoregulation [9], and hormonal regulation [10, 11]. Plants can improve their salt tolerance by increasing the activity of antioxidant enzymes and the expression of their related genes under salt stress [12, 13]. Osmoregulatory substances, such as proline, soluble sugars, and polyamines, can regulate plant adaptation to a variety of stresses by participating in osmoregulation [9, 14, 15]. Furthermore, plant hormones such as abscisic acid, ethylene, and jasmonic acid, known to participate in adaptive responses to various stresses, may regulate plant tolerance to salt stress [10, 11, 16].

Chrysanthemum (*Chrysanthemum morifolium*) is one of the top four cut flowers in the world and one of the top 10 traditional Chinese flowers [17, 18], and occupies an important position in the flower market [19–21]. Garden chrysanthemums, the most commercialized and industrialized ornamental chrysanthemums, are widely used for landscape creation in gardens, flowering seas, and urban street beautification. However, chrysanthemums are susceptible to salt stress in coastal mudflats and inland saline areas, leading to leaf shrinkage and plant withering. This seriously affects the growth and development of chrysanthemums and restricts their cultivation and application in coastal and inland saline areas.

Grafting, an ancient technique [22], is one of the most important methods for the vegetative propagation of horticultural crops, such as vegetables, fruits, and flowers [23, 24]. It has been used extensively in horticultural crops to improve their tolerance of biotic and abiotic stresses, to improve yields, and to modify plant shape [24–27]. Recently, grafting has been shown to improve aphid resistance [28, 29], heat tolerance [30], and drought resistance [31] in chrysanthemums. However, it remains unclear how heterologous rootstocks regulate chrysanthemum resistance under salt stress and how grafted and non-grafted chrysanthemums differ in their response to salt stress.

In the current study, we found that heterografted (HG) chrysanthemum plants (*Artemisia* as rootstocks) were more salt-tolerant than self-grafted (SG) and self-rooted (SR) chrysanthemum plants. Our results provide evidence, from both physiological and transcriptomic perspectives, that heterologous grafted chrysanthemums enhance salt tolerance through changes in reactive oxygen species (ROS), soluble sugar, and proline pathways. This will help us to understand the mechanisms by which grafting improves the salt tolerance of chrysanthemums, making it possible to cultivate them on a large scale in saline and coastal areas.

## Results

### Heterografted plants display significantly enhanced salt tolerance compared with self-grafted and self-rooted plants

**Figure 1:**
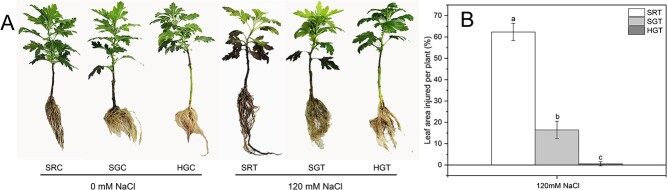
HG chrysanthemum plants display significantly enhanced salt tolerance compared with SR and SG chrysanthemum plants. **a** Phenotypes. **b** Leaf area injured per plant. The data in (**a**) and (**b**) were obtained after 8 days of salt treatment. The data were obtained under 0 mM NaCI (C) or 120 mM NaCI (T) treatment and are means of three replicates ± standard deviations. Means marked with different lower-case letters differed significantly at *P* < .05

To determine the effect of grafting on the salt tolerance of chrysanthemums, salt stress treatments were applied to SR, SG, and HG chrysanthemum plants. After 8 days of 120 mM sodium chloride (NaCl) treatment, SR plants showed a phenotype in which most of the leaves were damaged and the roots were browned (Fig. 1a and b). SG plants showed a phenotype of mild leaf damage and slightly browned roots (Fig. 1a and b). HG plants showed a healthier phenotype (Fig. 1a) with significantly lower leaf and root damage (Fig. 1b) than that in SG and SR plants. Together, these results show that HG plants are significantly more salt-tolerant than SG and SR plants.

### The roots of heterografted plants enriched Na^+^ to reduce Na^+^ content of leaves

**Figure 2:**
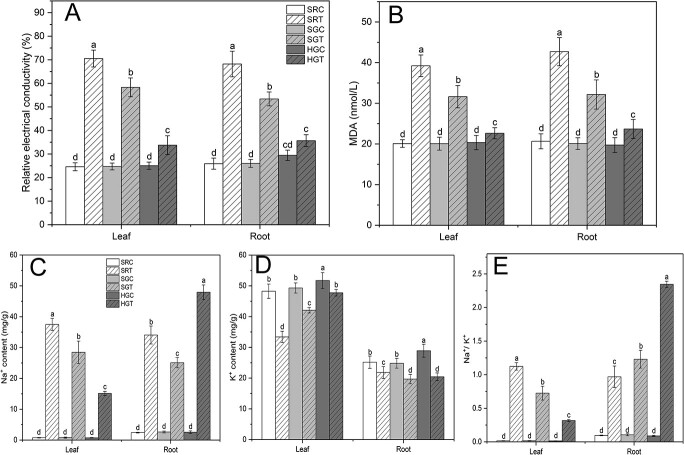
Roots of HG plants enriched Na^+^ to reduce the Na^+^ content of leaves. **a** Relative electrical conductivity. **b** MDA content. **c** Na^+^ content. **d** K^+^ content. **e** Na^+^/K^+^. Plants were treated as described in Fig. 1. Roots and leaves were sampled after 8 days of salt stress. Data were obtained under 0 mM NaCI (C) or 120 mM NaCI (T) treatment. Data are means of three replicates (± standard deviations), and means marked with different lower-case letters differed significantly at *P* < .05.

We also measured the relative electrical conductivity (REC), malondialdehyde (MDA) content, Na^+^, K^+^, and Na^+^/K^+^ to study the physiological changes in SR, SG, and HG plants. At 0 mM NaCI, no significant differences were found between these plants, except for a slightly higher K^+^ content in HG plants than in SG and SR plants. However, after 8 days of salt treatment, significant differences were observed between these plants in terms of REC and MDA content. Compared with the control, SR plants showed the highest REC and MDA, followed by SG plants and then HG plants (Fig. 2a and b). These results indicate that membrane lipid peroxidation in the leaves and roots of HG plants was significantly lower than that in SR and SG plants under salt stress.

Under salt stress, there were significant differences between plants in terms of Na^+^, K^+^, and Na^+^/K^+^. After 8 days of salt treatment, the Na^+^ content in the leaves of SR and SG plants was nearly 2.6- and 2.0-fold higher than that in HG plants, respectively. Surprisingly, the Na^+^ content in the roots of SR and SG plants was nearly 1.3- and 1.8-fold lower than that in HG plants, respectively. HG plants contained the most Na^+^ in the roots and the least Na^+^ in the leaves (Fig. 2c). The K^+^ content in the leaves and roots of SR, SG, and HG plants was significantly reduced after salt treatment compared with the control. The K^+^ content in the leaves was highest in HG plants, followed by SG plants, and lowest in SR plants. The K^+^ content of the roots was the same in HG plants as in SG plants and the lowest in SR plants (Fig. 2d). The trends of Na^+^/K^+^ versus Na^+^ content in the leaves and roots of SR, SG, and HG plants were similar after salt treatment, with the lowest values of Na^+^/K^+^ in the leaves of HG plants and the largest values of Na^+^/K^+^ in the roots of HG plants (Fig. 2e). These results indicate that HG plants enriched Na^+^ in the roots of rootstock, significantly reducing the content of Na^+^ in chrysanthemum leaves to alleviate Na^+^ damage to the leaves.

### Transcriptome sequencing and assembly results

RNAs were extracted from SR, SG, and HG chrysanthemum plants with no salt treatment (C) or with salt stress treatment (T). For this study we constructed 12 libraries, including leaf samples from the control group (L_SRC, L_SGC, L_HGC), root samples from the control group (R_SRC, R_SGC, R_HGC), leaf samples from the salt treatment group (L_SRT, L_SGT, L_HGT), and root samples from the salt treatment group (R_SRT, R_SGT, R_HGT). The summary statistics of the original and filtered clean reads are shown in Supplementary Data Table S1.

**Figure 3:**
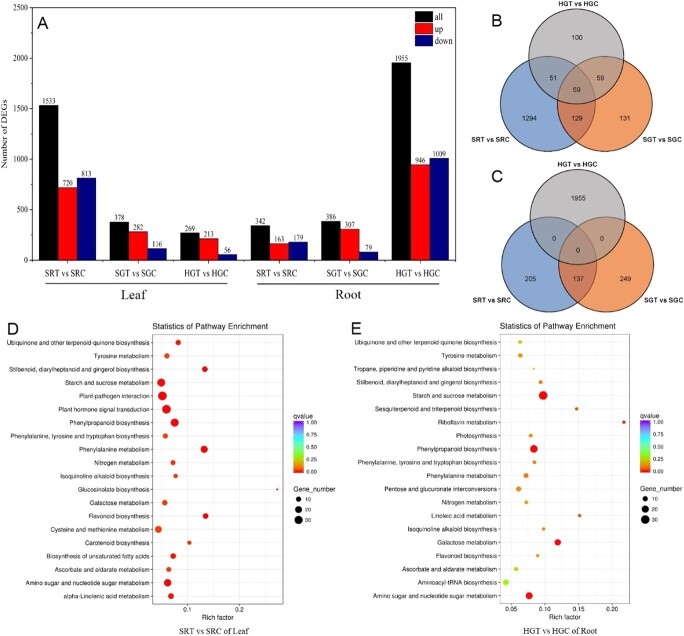
Statistical and KEGG enrichment analyses of DEGs. **a** Statistical analysis of upregulated and downregulated DEGs. **b** Venn diagrams of DEGs in the leaf. **c** Venn diagrams of DEGs in the root. **d** KEGG enrichment analysis of DEGs in SRT versus SRC of the leaf. **e** KEGG enrichment analysis of DEGs in HGT versus HGC of the root. Data were obtained under 0 mM NaCI (C) or 120 mM NaCI (T) treatment.

The clean reads of leaves from the SR, SG, and HG plants without salt treatment were between 56 358 088 and 57 664 590; the average clean reads of leaves from the SG, SG, and HG plants with salt stress treatment were between 49 376 046 and 55 220 900; the average clean reads of roots from the SG, SG, and HG plants without salt stress treatment were between 53 422 924 and 70 647 872; the average clean reads of roots from the SG, SG, and HG plants without salt stress treatment were between 43 899 190 and 53 955 386 (Supplementary Data Table S1). Among the 12 clean-read libraries, Q20 was >97.81% and Q30 was >93.76% (Supplementary Data Table S1). Then, by splicing clean reads, we obtained a reference sequence for subsequent analysis. The total number of transcripts was 733 546 (Supplementary Data Table S2), and the total number of unigenes was 266 749 (Supplementary Data Table S2). Then, all unigenes were compared with the seven major functional databases for annotation, and there were 62 018 genes annotated in the Kyoto Encyclopedia of Genes and Genomes orthology (KO) database (23.25%), 115 394 genes annotated in the gene ontology (GO) database (43.26%), 53 436 genes annotated in the EuKaryotic Orthologous Groups (KOG) database (20.03%), 127 572 genes annotated in the non-redundant (NR) database (47.82%), 83 639 genes annotated in the NT database (31.35%), 115 404 genes annotated in the SwissProt database (43.26%), and 115 394 genes annotated in the Pfam database (43.26%) (Supplementary Data Table S3).

### Differentially expressed genes in response to salt stress in self-rooted, self-grafted, and heterografted plants

We compared the differentially expressed genes (DEGs) among the 12 groups of samples (Supplementary Data Tables S4 and S5). As shown in Fig. 3a, there were 1533 DEGs (720 upregulated and 813 downregulated) in L_SRT compared with L_SRC; 378 DEGs (282 upregulated; 116 downregulated) in L_SGT compared with L_SGC; 269 DEGs (213 upregulated; 56 downregulated) in L_HGT compared with L_HGC; 342 DEGs (163 upregulated; 179 downregulated) in R_SRT compared with R_SRC; 386 DEGs (307 upregulated; 79 downregulated) in R_SGT compared with R_SGC; and 1955 DEGs (946 upregulated; 1009 downregulated) in R_HGT compared with R_HGC. From the leaf Venn diagram, there were 59 DEGs in SR, SG, and HG plants, 51 DEGs in SR and HG plants, 59 DEGs in SG and HG plants, 129 DEGs in SR and SG plants, and 1294, 131, and 100 DEGs only in SR, SG, and HG plants, respectively (Fig. 3b). From the root Venn diagram, 137 DEGs were identified in SR and SG plants, and 205, 249, and 1955 DEGs were identified only in SR, SG, and HG plants, respectively (Fig. 2c). To facilitate our analysis of the DEGs, we mapped the pathways of these DEGs using KEGG (Kyoto Enclyclopedia of Genes and Genomes). Results showed that DEGs in the leaves of SR plants were mainly enriched in plant hormone signal transduction (Ko04075), plant–pathogen interactions (Ko04626), starch and sugar metabolism (Ko00500), phenylpropanoid biosynthesis (Ko00940), and amino sugar and nucleotide sugar metabolism (Ko00520) (Fig. 3d). DEGs in the roots of HG plants were mainly enriched in starch and sugar metabolism, phenylpropanoid biosynthesis (Ko00940), and amino sugar and nucleotide sugar metabolism (Ko00520) (Fig. 3e).

The above results indicate that there were a large number of DEGs in the leaves of SR plants, including plant–pathogen interactions, starch and sugar metabolism, phenylpropanoid biosynthesis, and amino sugar and nucleotide sugar metabolism, to resist salt stress, but fewer genes were differentially expressed in the leaves of SG and HG plants. A large number of genes in the pathways of starch and sugar metabolism, phenylpropanoid biosynthesis, and amino sugar and nucleotide sugar metabolism were differentially expressed in the roots of HG plants to resist salt stress injury, but fewer genes were differentially expressed in the roots of SG and SR plants.

### Differentially expressed genes related to reactive oxygen species, Ca^2+^, and heat shock proteins

**Figure 4:**
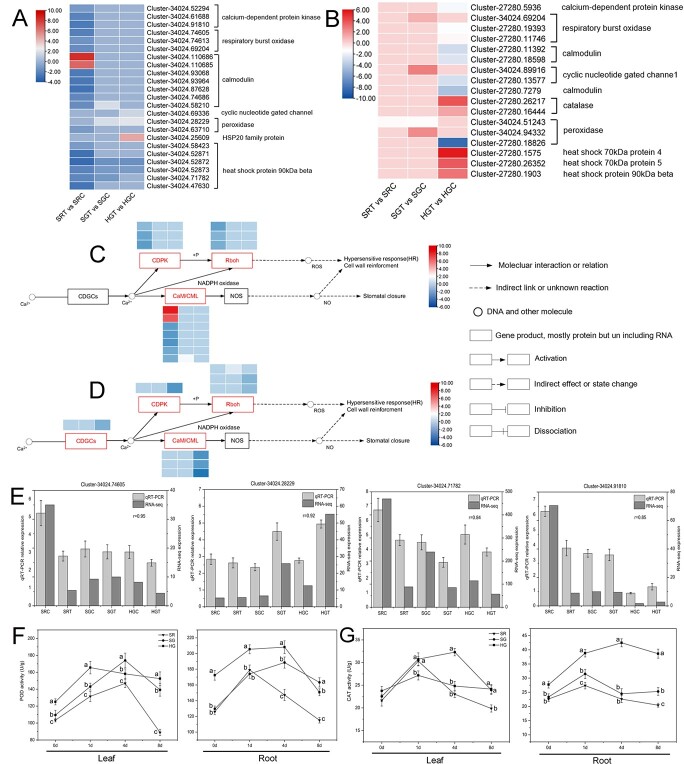
DEGs related to ROS and Ca^2+^. **a** Heat map of leaf DEGs related to ROS and Ca^2+^. **b** Heat map of root DEGs related to ROS and Ca^2+^. **c** Regulated pathway analysis of DEGs related to ROS and Ca^2+^ in the leaves of SR, SG, and HG plants. **d** Regulated pathway analysis of DEGs related to ROS and Ca^2+^ in the roots of SR, SG, and HG plants. The three columns of the heat map represent SR, SG, and HG plants from left to right. **e** Verification of the expression of four DEGs from (**a**) by qRT–PCR. **f** POD activity in leaves and roots of SR, SG, and HG plants. **g** CAT activity in leaves and roots of SR, SG, and HG plants. Roots and leaves were sampled after 0, 1, 4, and 8 days of salt stress. Data were obtained under 0 mM NaCI (C) or 120 mM NaCI (T) treatment. Data are means of three replicates (± standard deviations), and means marked with different lower-case letters differed significantly at *P* < .05.

ROS, Ca^2+^, and heat shock proteins (HSPs) play an important role in plant resistance to adversity and adaptation to the environment [32–34]. Our KEGG enrichment analysis showed that many genes were enriched in the plant–pathogen interaction pathway, which contains a large number of DEGs related to ROS, Ca^2+^, and HSPs. To clarify the changes in the resistance of grafted chrysanthemums to salt stress, we compared the expression of these genes in SR, SG, and HG plants after salt treatment. As shown in the leaf results (Fig. 4a), several calcium-dependent protein kinase (*CDPK*), respiratory burst oxidase (*RBOH*), and calmodulin (*CALM*) family genes were downregulated or upregulated in the leaves of SR plants under 8 days of salt stress treatment, but there were fewer DEGs in the leaves of SG and HG plants. A peroxidase (*POD*) family gene (Cluster 34024.63710) was downregulated in the leaves of SR plants and another *POD* family gene (Cluster 34024.28229) was upregulated in the leaves of SG and HG plants (Fig. 4a). In addition, several HSP 90-kDa β (*HSP90B*) family genes were downregulated in the leaves of SR, SG, and HG plants, but, interestingly, an *HSP20* family gene (Cluster 34024.25609) was significantly upregulated in the leaves of HG plants (Fig. 4a). As shown in the root results (Fig. 4b), several *CDPK*, *RBOH*, *CNGC*, and *CALM* family genes were significantly downregulated in the roots of HG plants under 8 days of salt stress treatment, but the DEGs of the roots in SR and SG plants were lower. Surprisingly, a *POD* family gene (Cluster 27280.18826) was significantly downregulated in the roots of HG plants, but several catalase (*CAT*) family genes (Cluster 27280.26217, Cluster 27280.16444) were significantly upregulated in the roots of HG plants. Furthermore, an *HSPA4* family gene (Cluster 27280.1575), an *HSPA5* family gene (Cluster 27280.26352), and an *HSP90B* family gene (Cluster 27280.1903) were significantly upregulated in the roots of HG plants (Fig. 4b). From the regulated pathway (Fig. 4c and d), we found that the downregulation of *CDPK*, *RBOH*, *CDGC*, and *CALM* genes resulted in reduction of ROS and NO production in the leaves. In addition, we verified the expression levels of four genes in the leaves of SR, SG, and HG plants by quantitative reverse transcription–polymerase chain reaction (qRT–PCR) assays, including the *RBOH* homologs (Cluster 34024.74605), *POD* homologs (Cluster 34024.28229), *HSP90B* homologs (Cluster 34024.71782), and *CDPK* homologs (Cluster 34024.91810) (Fig. 4e). The expression trends of these four genes were consistent with RNA sequencing data.

From Fig. 4f and g, we found that POD and CAT enzyme activities differed between different types of plants and different tissue parts. As the number of days of salt treatment increased, POD and CAT enzyme activities of the leaves and roots in the three types of plants increased and then decreased, reaching a maximum on the first or fourth day after treatment. The enzyme activities of POD and CAT in leaves were the highest in SG plants, followed by HG and SR plants. At 8 days of salt treatment, both enzyme activities in leaves of SG and HG plants were higher than before treatment (0 days), while both enzyme activities in leaves of SR plants were lower than control (0 days), and much lower than in SG plants and HG plants (Fig. 4f). Based on the enzyme activity of POD in the roots, the enzyme activity of HG plants at 0-4days of salt treatment was much higher than that before treatment, and much higher than that in SG and SR plants, but, surprisingly, the POD activity of HG plants was significantly lower than that before treatment (0 days) and that of SG plants at 8 days of salt treatment. Importantly, from the enzyme activity of CAT in the roots, the CAT activity in the roots of HG plants was maintained at a higher level after salt treatment; it was much higher than before treatment (0 days) and that in SG and SR plants (Fig. 4g). These results showed that ROS production-related genes were downregulated and ROS accumulation was reduced in HG plant roots; *CAT* family genes were significantly upregulated and ROS scavenging was accelerated; and *HSPA4*, *HSPA5*, and *HSP90B* family genes were significantly upregulated and plant stress resistance was enhanced.

### Heterografted plants accumulate more soluble sugars in response to salt stress

**Figure 5:**
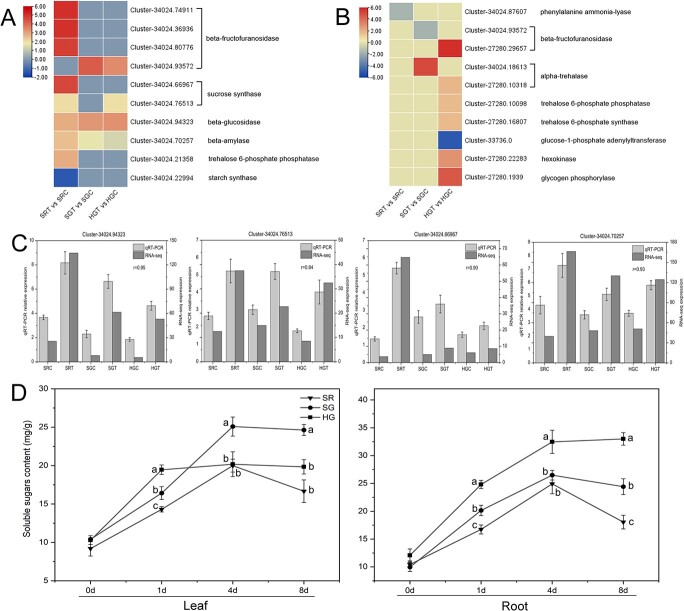
DEGs involved in starch and sucrose metabolism. **a** Heat map of leaf DEGs involved in starch and sucrose metabolism. **b** Heat map of root DEGs involved in starch and sucrose metabolism. **c** Verification of the expression of four DEGs in (**a**) by qRT–PCR. **d** Soluble sugar content in leaves and roots of SR, SG, and HG plants. Data were obtained under 0 mM NaCI (C) or 120 mM NaCI (T) treatment. Data are means of three replicates (± standard deviations), and means marked with different lower-case letters differed significantly at *P* < .05.

To determine whether soluble sugars were associated with increased salt tolerance in grafted chrysanthemums, we compared DEGs of starch and soluble sugar metabolic pathways in SR, SG, and HG plants before and after salt treatment. From the heat map in Fig. 5a and b, it was found that several β-fructofuranosidase (*INV*) genes, several sucrose synthase (*SUS*) genes and other genes related to starch and sucrose metabolism were significantly upregulated in the leaves of SR plants after 8 days of salt treatment. The expression of *INV*, *SUS*, β-glucosidase, and β-amylase was also significantly upregulated in the leaves of SG and HG plants (Fig. 5a). There were few DEGs related to starch and sucrose metabolism in the roots of SR and SG plants after 8 days of salt treatment, with only a β-glucosidase family gene downregulated in the roots of SR plants, an *INV* family gene downregulated, and an α-trehalase (*treA*) family gene was significantly upregulated in the roots of SG plants. Interestingly, a large number of starch and sucrose metabolism-related genes, such as *INV*, *treA*, trehalose 6-phosphate synthase (*otsA*), *otsB*, glycogen phosphorylase (*glgP*), and hexokinase (*HK*), were significantly upregulated in the roots of HG plants (Fig. 5b). The expression levels of four genes in the leaves of SR, SG, and HG plants were verified by qRT–PCR assays, including the β-glucosidase homologs (Cluster 34024.94323), *SUS* homologs (Cluster 34024.76513, Cluster 34024.66967), and β-amylase homologs (Cluster 34024.70257) (Fig. 5c), which is consistent with the RNA sequencing data.

**Figure 6:**
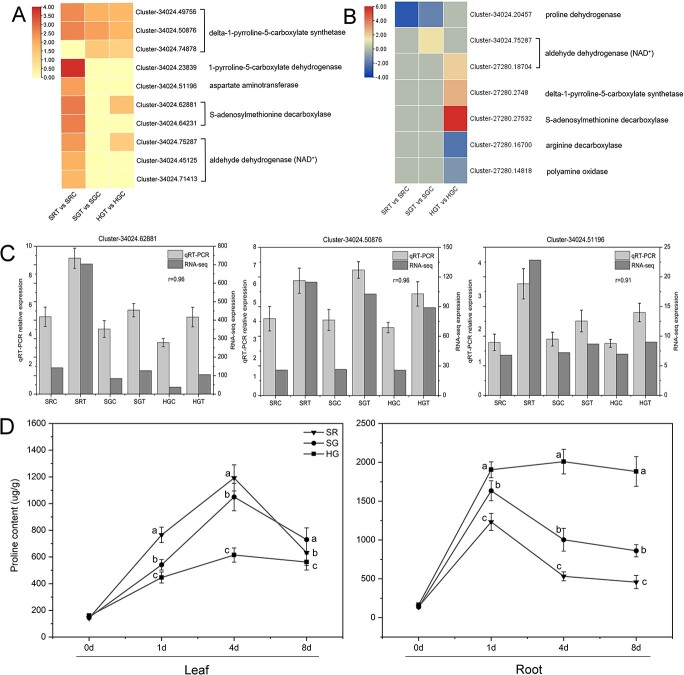
DEGs involved in proline and arginine metabolism. **a** Heat map of leaf DEGs involved in proline and arginine metabolism. **b** Heat map of root DEGs involved in proline and arginine metabolism. **c** Verification of the expression of three DEGs in (**a**) by qRT–PCR. Left vertical axis represents fragments per kilobase per million mapped fragments (FPKM) from RNA sequencing; right vertical axis represents relative gene expression level from qRT–PCR. *R*-values are the relative coefficients between qRT–PCR and RNA sequencing. **d** Proline content in leaves and roots of SR, SG, and HG plants. Data were obtained under 0 mM NaCI (C) or 120 mM NaCI (T) treatment. Data are means of three replicates (± standard deviations), and means marked with different lower-case letters differed significantly at *P* < .05.

We also found that the soluble sugar content in the leaves of SR, SG, and HG plants was significantly increased after 8 days of treatment (Fig. 5d). The highest soluble sugar content was found in the leaves of SG plants after 8 days of treatment, in line with the higher expression of multiple soluble sugar-related genes in SR plants. As shown in Fig. 5d, the soluble sugar content in the roots of SR, SG, and HG plants increased significantly after 8 days of treatment and were all higher than before treatment (0 days). Importantly, HG plants showed the highest soluble sugar accumulation. These results indicate that the expression of genes related to starch and sugar metabolism is significantly upregulated in the roots and leaves of HG plants, resulting in a significant accumulation of soluble sugars and improved salt stress tolerance.

### Heterografted plants accumulate more proline in response to salt stress

To determine the relationship between proline and salt tolerance in grafted chrysanthemums, we compared the DEGs of the proline and arginine metabolism pathways in SR, SG, and HG plants before and after salt treatment. In the leaves of SR plants after 8 days of salt treatment, two δ-1-pyrroline-5-carboxylate synthetase (*P5CS*) family genes, a 1-pyrroline-5-carboxylate dehydrogenase (*P5CDH*) family gene, two *S*-adenosylmethionine decarboxylase (*SAMDC*) family genes, three aldehyde dehydrogenase (*ALDH*) family genes, and an aspartate aminotransferase (*GOT1*) family gene were significantly upregulated (Fig. 6a). Three *P5CS* family genes were significantly upregulated in the leaves of the SG plants. Three *P5CS*, *ALDH*, and *SAMDC* family genes were upregulated in the leaves of the HG plants. *P5CS* is the rate-limiting enzyme in proline biosynthesis and determines the rate of proline accumulation in plants [35]. *P5CDH* is the rate-limiting enzyme in proline degradation [36]. Thus, after 8 days of salt stress, proline synthesis was greater than catabolism, and proline content increased in SR leaves, although both *P5CS* and *P5CDH* were upregulated, and proline accumulated in the leaves of SG and HG plants. In addition, we found that the proline content in the leaves of SR and SG plants was highest after 4 days of salt treatment (Fig. 6d) and significantly decreased after 8 days of treatment, but was still much higher than in control (0 days). The proline content in the leaves of HG plants increased slowly and remained at a higher level. This is consistent with the expression of *P5CS* and *P5CDH* family genes.

As shown in Fig. 6b, only the proline dehydrogenase (*PRODH*) family gene was significantly downregulated in the roots of SR plants. Only a *PRODH* family gene was downregulated in SG plant roots, and an *ALDH* family gene was significantly downregulated. *P5CS*, *ALDH*, and *SAMDC* family genes were significantly upregulated, and arginine decarboxylase (*ADC*) and polyamine oxidase (*PAO*) family genes were downregulated in the roots of HG plants. PRODH is an important enzyme in proline catabolism [37]. After 8 days of salt stress, the proline catabolic gene *PRODH* was downregulated in SR and SG roots, and several *P5CS* family genes were significantly upregulated in the roots of HG plants, resulting in the accumulation of proline. In addition, the highest proline content was found in the roots of SR and SG plants after 1 day of salt treatment, and it was decreased significantly on day 8 of treatment (Fig. 6d). However, the proline content in the roots of HG plant was found to have increased rapidly at the early stage of salt treatment, and then to decrease slowly with a longer time of treatment, resulting in a much higher level of proline accumulation in HG than in SR and SG plants. The results are largely consistent with the dynamic changes of gene expression in these plants. Furthermore, the expression levels of three selected genes were verified by qRT–PCR assays, including the *SAMDC* homologs (Cluster 34024.62881), *P5CS* homologs (Cluster 34024.50876), and *GOT1* (Cluster 34024.51196). These genes demonstrated expression patterns consistent with the expression levels obtained from RNA-seq analysis (Fig. 6c).

## Discussion

It is known that grafting a plant onto a resistant rootstock can enhance its tolerance to various environmental stresses, such as soil-borne diseases [38], drought [39, 40], cold [41], and salt stress [42]. In this study, we found that HG chrysanthemum plants were significantly more tolerant to salt stress than SR and SG chrysanthemum plants. This is similar to the findings of previous studies. Furthermore, we also found that the rootstock of HG chrysanthemum plants contained high levels of Na^+^ under salt stress, but the roots and leaves of the plants remained healthy by integrating the ROS, soluble sugar, and proline pathways. In the future, we can improve the salt tolerance of chrysanthemums by grafting, making it possible to cultivate them on a large scale in saline and coastal areas.

Salt stress is known to cause osmotic stress and ion toxicity, which severely restrict plant growth and development [1, 43, 44]. Both osmotic stress and ionic stress can lead to the accumulation of high levels of ROS, resulting in ROS toxicity [45, 46], including superoxide (O_2_^−^), hydrogen peroxide (H_2_O_2_), and hydroxyl radicals (OH^·–^), which induce cytoplasmic membrane damage and metabolic dysfunction [44, 47]. Thus, ROS scavenging enzymes in plants are directly related to ROS cytotoxicity [8]. Plants produce antioxidant defense systems, including superoxide dismutase (*SOD*), *POD*, *CAT*, and ascorbate peroxidase (*APX*), which inhibit oxidative damage by scavenging excess ROS [48, 49]. In this study, we found that two *CAT* family genes were significantly upregulated in HG plant roots compared with SG and SR plants, and a *POD* family gene was upregulated in the leaves, implying that HG plants exhibited greater ROS scavenging capacity. This is consistent with previous reports [41, 50, 51]. Surprisingly, we found that the POD activity of HG plant roots was significantly lower than that before treatment (0 days) at 8 days of salt treatment, which may be related to our subsequent finding of slowed ROS accumulation after 8 days of salt stress. ROS not only induce cytoplasmic membrane damage and metabolic dysfunction, but also as important signaling molecules , like Ca^2+^, in plant growth, development, and stress resistance [32, 33, 52]. NADPH oxidases in plants, also known as RBOHs, are located in the cytoplasmic membrane and can produce ROS in apoplasts [51, 53, 54]. CDPK and CALM are calcium-sensing proteins that have been reported to regulate the activity of RBOHs by phosphorylating them, thus regulating the level of ROS [51, 55, 56]. In this study, we also found that Ca^2+^ signaling-related genes, such as *CDPK*, *RBOH*, and *CALM*, were significantly downregulated in the roots of HG plant roots compared with SG and SR plants, which may have led to a decrease in ROS accumulation.

HSPs are regulators of cellular stress and play an important role in plant resistance to adversity and adaptation to the environment. We found that *HSPA4*, *HSPA5*, and *HSP90B* were significantly upregulated in the roots of HG plants compared with SG and SR plants, which is consistent with reports that *HSFA4* confers enhanced salt tolerance in chrysanthemum [45] and *Arabidopsis thaliana* [57] and that *HSP90B* is involved in plant stress tolerance processes [58, 59]. HSPs are associated with increased salt tolerance in HG chrysanthemums. In addition, although plant hormones play an important role in response to stress [10, 11, 16], we found that fewer hormone-related genes were significantly differentially expressed in the roots and leaves of HG plants and that the correlation between the DEGs of hormone signals and enhanced salt stress tolerance in HG plants was low, possibly due to species variability.

Starch and sucrose metabolism determine the levels of soluble sugars, which are important osmoregulatory substances that alleviate osmotic stress and have been shown to play a crucial role in plant salt tolerance [14, 60, 61]. In this study, we found that a large number of starch and sugar metabolism genes were significantly upregulated in SR plant leaves, and several genes were upregulated in SG and HG plant leaves, with the highest soluble sugar content in SG plant leaves, followed by HG and SR plants. This is consistent with previous studies that found that heterologous grafting accelerated the synthesis of soluble sugars in chrysanthemum leaves and enhanced aphid resistance in chrysanthemums [28]. Importantly, we also found that a large number of starch and sugar metabolism genes were significantly upregulated in HG plant roots and that soluble sugar content was significantly increased in HG plant roots under salt stress, compared with SG and SR plants. This means that although all three plant types respond to osmotic stress through the upregulation of starch and sugar metabolism genes under salt stress, the extent of upregulation and increase in soluble sugar content in HG plant roots was most pronounced. This resulted in a large accumulation of soluble sugars in the roots of HG plants, which alleviated the osmotic stress caused by high Na^+^ concentrations and showed salt tolerance.

**Figure 7:**
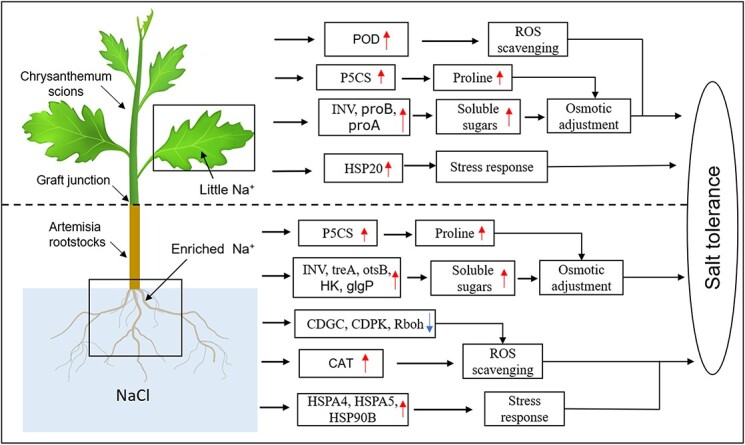
A model for mechanisms underlying the enhanced salt tolerance of HG chrysanthemum plants.

As an osmoregulatory substance in plant cells, proline is widely present in higher plants and is usually accumulated in large quantities under environmental stress [62–65]. In addition to increasing the osmotic potential of plant cells and improving plant stress tolerance, proline also helps to stabilize subcellular structures, scavenge free radicals, and buffer the cellular redox potential under stress conditions [66, 67]. In this study, after 8 days of salt stress, several *P5CS* family genes were upregulated in the leaves of SR, SG, and HG plants, and proline content was significantly increased, but the proline content in the leaves of HG plants was slightly lower than that in SR and SG plants. This is consistent with previous studies [68], and, as for the lower proline content in leaves of HG plants compared with SR and SG plants, it may be related to the lower Na^+^ concentration and lower stress level in the leaves of HG plants. Notably, compared with SR and SG plants, the *P5CS* family genes were significantly upregulated in the roots of HG plants, resulting in a significant increase in proline content. This means that proline accumulates in large quantities in the roots of HG plants, relieving the toxic effects caused by high concentrations of Na^+^. In addition, we also found that *SAMDC* family genes were upregulated in the roots and leaves of HG plants, and arginine decarboxylase (*ADC*) and polyamine oxidase (*PAO*) family genes were downregulated in the roots of HG plants. These three enzymes are key to the synthesis and metabolism of polyamines [69], which are important regulators of plant adaptation to adversity [70, 71] and are involved in the regulation of salt tolerance in plants [72–74], implying that polyamines may be involved in the improvement of salt tolerance in HG plants.

In conclusion, our study revealed the mechanisms by which HG plants exhibit increased salt tolerance relative to SG and SR plants (Fig. 7). Under salt stress, the roots of HG plants are enriched in Na^+^, leading to a small amount of Na^+^ being transported to the leaves of HG plants. The roots of HG plants alleviated the toxic effects of high Na^+^ concentrations and enhanced salt tolerance via reduced ROS accumulation, significantly increased CAT activity to enhance the ability of ROS scavenging, upregulated the expression of genes related to HSPs to improve stress tolerance, and significantly accumulated soluble sugars and proline. In response to low-intensity Na^+^ stress and to maintain a healthy state, the leaves of HG plants exhibited higher soluble sugar and proline content, and increased POD activity to scavenge ROS, and upregulated the expression of *HSP20* family genes to improve stress tolerance. However, SG and SR plants do not have the capacity to integrate ROS, soluble sugars, and proline in response to salt stress, and thus exhibit a salt-sensitive phenotype. In summary, the current study reveals the physiological and molecular mechanisms underlying the increased salt tolerance of HG chrysanthemums, facilitating the large-scale cultivation of chrysanthemums in saline areas.

## Materials and methods

### Plant material and growth conditions

*Artemisia annua* (Aa) and the garden chrysanthemum cultivar ‘Zhongshan Yanhong’ (Cm) were obtained from the Chrysanthemum Germplasm Resource Conservation Center, Nanjing Agricultural University, China. Aa was propagated by seeds and Cm by cutting. When the stems of Aa and Cm were 5 mm thick, some Cm cuttings were selected as self-rooted plants, and Aa and Cm were used as scions or rootstocks for grafting by cleft-grafting to obtain Cm/Cm [self-grafted (SG)] plants and Cm/Aa [heterografted (HG)] plants, respectively. SR, SG, and HG plants were used for subsequent trials. All plants were grown in a greenhouse with a mean temperature of 25/18°C (day/night) and a 14-h light/10-h dark photoperiod, with a relative humidity of 60–70%.

### Salt stress treatment and plant sampling

Thirty days after grafting, the three types of plants (SR, SG, and HG) were transferred to a plastic crate (volume 23.4 l) containing 1/2 Hoagland nutrient solution for slow-growing hydroponics and aerated for 24 hours/day. Before the salt treatment, we set four concentrations of NaCl at 0, 80, 120 and 160 mM for the scion (*Chrysanthemum*) and rootstock (*A. annua*) to screen for the best concentration for salt treatment. We found slight rootstock damage and heavy scion damage at the concentration of 120 mM; however, no significant damage differences between scion and rootstock were observed at the concentrations of 80 and 160 mM. We selected 120 mM NaCl for this study. After 7 days of preculture in hydroponics, the three types of plants were treated at 0 mM NaCl (control) and 120 mM NaCl (treatment). Leaves and roots were taken from plants at 0, 1, 4, and 8 days and stored at −80°C to determine physiological indicators. When sampling, only the third mature leaves from the top of the plant were taken, while the entire root system was taken from the plant. After 8 days of salt treatment, the third mature leaves from the top and the entire root system were collected for RNA extraction and transcriptome sequencing. For each treatment, six plants were mixed into 1 sample for a total of 12 samples.

### Physiological measurements

ImageJ software (https://imagej.nih.gov/ij/) was used to measure the total leaf area per plant and the injured area of leaves per plant, and then to calculate the leaf area per plant. REC was measured using the leaf immersion method [75]. After overnight acidification with nitric acid (HNO_3_), Na^+^ and K^+^ concentrations were measured using 0.2 g of leaf and root samples following a reported method [76]. The activities of POD, SOD, and CAT, as well as the contents of soluble sugars, MDA, and proline, were analyzed using an enzyme-linked immunosorbent assay (ELISA) kit (Nanjing Jiancheng Bioengineering Institute, Nanjing, China) following the manufacturer’s instructions. Each determination included three biological and technical replicates.

### RNA extraction and transcriptome sequencing

The total RNA was isolated from 12 samples using RNAiso Plus reagent (TaKaRa, Tokyo, Japan) under the manufacturer’s protocol. We assessed RNA integrity by agarose gel electrophoresis and the measurement of RNA concentration. The mRNA from each sample was purified, fragmented, and transcribed to generate sequencing libraries. End-repair and 3′-adenylation of the cDNA were performed. The ends of those cDNA fragments that were 3′-adenylated were attached to NEBNext adapters with a hairpin loop structure. Next, the fragments mentioned above were purified and cDNA fragments 250–300 bp in length were selected for PCR. The PCR products were purified and evaluated. An Illumina HiSeq platform was used to sequence the library and generate the paired-end reads. There were 12 groups of raw readings, including leaf samples from the control group (L_SRC, L_SGC, L_HGC), root samples from the control group (R_SRC, R_SGC, R_HGC), leaf samples from the salt treatment group (L_SRT, L_SGT, L_HGT), and root samples from the salt treatment group (R_SRT, R_SGT, R_HGT).

### Transcriptome assembly and differentially expressed gene function annotation

Reference sequences were reconstructed using Trinity [77]. First, the original sequencing reads were filtered to remove low-quality reads, producing clean reads. The clean reads were assembled using Trinity. The transcripts obtained were then used as reference sequences for analysis. Single gene expression levels were computed as fragments mapped per kilobase of transcript per million fragments (FPKM) [78]. Pearson’s correlation method was used to compute the correlation between the two samples. Differential expression analysis between the two samples was conducted using the DEGseq R package [79, 80]. We used *Q* to adjust *P* values [81]. The criteria for selecting genes that were significantly differentially expressed were |Log_2_ (FoldChange)| > 1 and *q* value <.005. The transcript sequences were matched with the following databases: NR (ftp://ftp.ncbi.nlm.nih.gov/blast/db), Swiss-Prot (www.uniprot.org), NT (ftp://ftp.ncbi.nlm.nih.gov/-blast/db), KEGG (http://www.genome.jp/kegg), GO (http://geneontology.org), Pfam (http://pfam.xfam.org), and KOG (https://www. ncbi.nlm.nih.gov/COG/). Next, a range of genes were determined and annotated. We used KEGG to map the sequences to the pathways [82], and DEG statistical enrichment found in the KEGG pathway was further tested using the KOBAS software [83]. A *q*-value ≤.05 was used to screen for significantly enriched pathways.

### Validation of RNA-seq data by qRT–PCR

In this study, 11 key genes were selected to verify the consistency of their expression pattern. Reverse transcription of total RNA (1 mg) was performed using Prime Script™ RT Master Mix (Perfect Real Time) (Takara) following the manufacturer’s instructions. Gene-specific primer pairs for the selected genes were designed using primer design software (Primer Premier 5) and *CmEF1α* was used as a reference (Supplementary Data Table S6). Each sample was studied in three biological replicates and qRT–PCR assays were performed as previously reported [84]. The relative expression levels of genes were computed using the 2^−ΔΔCT^ method [85].

## Supplementary Material

Web_Material_uhac073

## Data Availability

The sequence data were submitted to the National Center for Biotechnology Information (NCBI; https://www.ncbi.nlm.nih.gov/, accessed on 1 February 2023) database under accession number SUB10946175. All other data included in this study are available from the corresponding author on reasonable request.
